# Hashimoto's Thyroiditis: From Genes to the Disease 

**DOI:** 10.2174/138920211798120763

**Published:** 2011-12

**Authors:** Katja Zaletel, Simona Gaberšček

**Affiliations:** Department of Nuclear Medicine, University Medical Centre Ljubljana, Ljubljana, Slovenia

**Keywords:** Endogenous factors, environmental factors, genetic susceptibility, Hashimoto’s thyroiditis, self-tolerance, thyroid destruction.

## Abstract

Hashimoto’s thyroiditis (HT) is the most prevalent autoimmune thyroid disorder. Intrathyroidal lymphocytic infiltration is followed by a gradual destruction of the thyroid gland which may lead to subclinical or overt hypothyroidism. Biochemical markers of the disease are thyroid peroxidase and/or thyroglobulin autoantibodies in the serum which are present with a higher prevalence in females than in males and increase with age. Although exact mechanisms of aetiology and pathogenesis of the disorder are not completely understood, a strong genetic susceptibility to the disease has been confirmed predominantly by family and twin studies. Several genes were shown to be associated with the disease occurrence, progression, and severity. Genes for human leukocyte antigen, cytotoxic T lymphocyte antigen-4, protein tyrosine phosphatase nonreceptor-type 22, thyroglobulin, vitamin D receptor, and cytokines are considered to be of utmost importance. Amongst endogenous factors for the disease development, the attention is focused predominantly on female sex, pregnancy with postpartum period and fetal microchimerism. Environmental factors influencing HT development are iodine intake, drugs, infections and different chemicals. Disturbed self-tolerance accompanied by the increased antigen presentation is a prerequisite for the HT occurrence, whereas proper interaction of thyroid cells, antigen presenting cells, and T cells are necessary for the initiation of thyroid autoimmunity. Secreted cytokines lead predominantly to T-helper type 1 (Th1) response as well as to Th 17 response which has only recently been implicated. Final outcome of HT is thyroid destruction which is mostly a consequence of the apoptotic processes combined with T-cell mediated cytotoxicity.

## INTRODUCTION

Hashimoto’s thyroiditis (HT) is the most prevalent autoimmune thyroid disorder, where lymphocytic infiltration of the thyroid gland is often followed by a gradual destruction and fibrous replacement of the thyroid parenchymal tissue. Patients may or may not develop a goitre. The principal biochemical characteristic of the disease is the presence of thyroid autoantibodies (TAbs) in the patients’ sera against two major thyroid antigens, thyroid peroxidase (TPO) and thyroglobulin (Tg). TPO antigen, located at the apical membrane of the thyrocyte, is essential for thyroid hormone synthesis, catalysis of iodine oxidation, iodination of tyrosine residues in Tg and coupling of the iodothyrosines into thyroxine (T_4_) and triiodothyronine (T_3_). The thyroid hormones are synthesized on Tg, a large glycoprotein within thyroid follicles, which also serves as the storage for thyroid hormones [1]. Small amount of Tg is secreted into the circulation where the estimated half-life is approximately 3 days [2].

Antibodies against TPO (TPOAbs) and Tg (TgAbs) are of immunoglobulin G class, both showing high affinity for their respective antigens. Unlike TgAbs, TPOAbs can activate complement and are able to cause damage to thyroid cells due to antibody dependent cell cytotoxicity [3]. Nevertheless, there is little evidence that both antibodies have a prime role in the pathogenesis of HT and it is far more likely that both T-cell mediated cytotoxicity and activation of apoptotic pathways influence the disease outcome. However, TAbs serve as a useful marker for the diagnosis of thyroid autoimmunity. In HT, TPOAbs are present in nearly all (>90 %) patients, while TgAbs can be detected in approximately 80% [1, 3]. 

The prevalence of HT confirmed by cytology was 13.4% in consecutive patients who underwent fine-needle aspiration biopsy of thyroid nodules and was similar to that of type 2 diabetes [4]. Beside lymphoid follicles, changes in epithelial cells, formation of connective tissue, and diffuse round cell infiltration, in his report Hashimoto described also some cracking spaces close to lymphoid follicles. It is now known that these cracking spaces are mainly lymphatic vessels, localized within the interlobular septa. Their number increases within the thyroid parenchyma near the lymphoid follicles [5].

The clinical disease may present with a variety of different manifestations ranging from a simple TAbs presence in patients with normal thyroid function to the development of severe thyroid dysfunction. Some patients present with short periods of mild thyrotoxicosis which usually cease spontaneously. Most often, euthyroid phase is followed by a gradual development of subclinical hypothyroidism which progresses slowly to overt hypothyroidism with the estimated annual risk of 4% in females [6]. According to large epidemiological surveys, HT is the most frequent cause of hypothyroidism recorded in 4% to 9.5% of the adult population [7-10]. The prevalence of HT is high which was also confirmed by the largest National Health and Nutrition Examination Survey (NHANES) III study. The results show that 18% of the population without previously known thyroid disease regardless age or gender presented with elevated TAbs; TPOAbs were positive in 11.3% and TgAbs in 10.4%. The prevalence of TAbs in females was twice as high as in males. It increased with age and was significantly higher in whites or Japanese than in blacks or Mexican Americans [8, 11]. Thus, approximately 20% of females older than 60 years were TAbs positive [8]. 

In spite of a very high HT prevalence, the exact mechanisms responsible for the disease development are still not completely understood. However, in the last decade there has been a significant advancement in the knowledge of the aetiology and pathogenesis of autoimmune thyroid disease which most frequently occurs in the form of HT or Graves’ disease (GD). In this review we discuss the current evidence of the possible triggers provoking HT in susceptible individuals and putative mechanisms leading to thyroid destruction in HT patients.

## GENETIC SUSCEPTIBILITY

### Epidemiological Evidence

For several decades a strong genetic predisposition to autoimmune thyroid disease has been recognised, predominantly on the basis of the family and twin studies. Nearly 50 years ago, soon after the discovery of TAbs, the presence of TAbs was reported in 56% of siblings of patients with autoimmune thyroid disease [12]. This familial clustering of autoimmune thyroid disease and the presence of TAbs in up to 60% of first-degree relatives of patients has been later confirmed by several studies [13-17]. When both parents were affected, the prevalence of TPOAbs and TgAbs was 42% in daughters and 33% in sons, compared with 28.9% and 16.7%, respectively, when only one parent was TAb-positive [14]. Among first-degree relatives of children with HT, 34% were diagnosed TPOAbs positive compared to only 13% first-degree relatives of children without autoimmune thyroid disease [17]. The sibling risk ratio for HT, calculated on the basis of the data from the NHANES III study, was 28, thus confirming the highly significant contribution of genetic factors to the disease development [18]. Recent data from Germany also indicate 32-fold increased risk for developing HT in children and 21-fold increased risk in siblings of patients with HT, with females being significantly more often affected than males [19]. 

Twin studies provided further valuable data on the genetic contribution to thyroid autoimmunity. In healthy twin siblings of patients with overt autoimmune thyroid disease, positive TPOAbs and TgAbs in monozygotic twins were determined in 53% and 47%, respectively, in dizygotic twins in 22% and 13%, respectively, while in healthy control population only in 9% and 7%, respectively [20]. The concordance rates for TPOAbs were 64% in monozygotic twins compared with 35% in dizygotic twins, while concordance rates for TgAbs were 74% and 32%, respectively [21]. The concordant rate for overt Hashimoto’s hypothyroidism was 55% in monozygotic twins and 0% in dizygotic twins [22], indicating the importance of non-genetic influences on the disease development. As assessed by a study of Danish twins, 73% of the susceptibility to the development of TAbs seems to be attributable to the genetic factors [23]. Moreover, a recent twin study indicated that the liability to the production of antibodies directed against immunodominant region A of TPO is genetically determined [24]. 

### Susceptibility Genes

#### Human Leukocyte Antigen (HLA) Genes

The first gene locus identified in association with the autoimmune thyroid disease was major histocompatibility complex (MHC) region on the chromosome 6p21 which encodes human leukocyte antigens (HLAs). HLA region, which is highly polymorphic, comprises several immune response genes. HLA molecule, located on antigen presenting cell (APC), binds and presents an antigenic peptide and in this way enables T cell recognition and response to an antigen. Presumably, specific HLA alleles have a higher affinity for autoantigenic thyroidal peptides and are thus likely to contribute to the development of the autoimmune thyroid disease. Nevertheless, in order to initiate the thyroid autoimmunity autoantigen occurrence within thyroid or thyroid draining lymph nodes is needed, being followed by HLA presentation. In HT, aberrant expression of HLA class II molecules on thyrocytes has been demonstrated. Presumably, such thyrocytes may act as APCs capable of presenting the thyroid autoantigens and initiating autoimmune thyroid disease [25]. 

While in GD, *HLA-DR3* was reported by several studies as primary susceptibility allele, no consistent associations were observed in HT. In Caucasians, associations of different forms of HT with various *HLA* alleles were reported, including *DR3* [26], *DR5*, *DQ7* [27], DQB1*03 [28], *DQw7* [29] or DRB1*04-DQB1*0301 haplotype [30]. In Japanese, associations with DRB4*0101, *HLA-A2* [31] and *DRw53* [32] were demonstrated, while in Chinese patients association with *DRw9* was observed [33].

#### Cytotoxic T Lymphocyte Antigen-4 (CTLA-4) Gene

*CTLA-4* gene, which is the second major immune-regulatory gene related to autoimmune thyroid disease, lies on chromosome 2q33. The expression of CTLA-4 on the surface of T cells, induced by the activation of the T-cell receptor, results in suppression of T-cell activation. *CTLA-4* gene polymorphisms may reduce expression or function of the CTLA-4 antigen and may therefore contribute to the reduced inhibition of T-cell proliferation and subsequently increase susceptibility to autoimmune response. In the past, several polymorphisms of the *CTLA-4* gene in HT patients were studied. Among them, the initially reported (AT)n microsatellite *CTLA-4* polymorphism in the 3’ untranslated region (UTR) was found to be associated with HT in Caucasian [34] and Japanese patients [35], but not in Italian population [30]. In the exon 1 located 49A/G single nucleotide polymorphism (SNP), resulting in threonine to alanine substitution, was associated with HT [36-38], however, certain other studies have not confirmed this observation [30, 39-41]. A large meta-analysis, including both published and unpublished data of 866 HT patients, indicated a significant association with 49A/G (summary OR 1.29; 95% CI, 1.11-1.50) [42]. Another *CTLA-4* polymorphism is 6230A/G SNP which is located at 3’-UTR and designated *CT60* [43]. Initial observation of the association with HT [43, 44] was not confirmed by later studies, including ours [41, 45], however, the results of the meta-analysis, based on six published and unpublished studies of 839 HT patients, indicated a significant association with *CT60* SNP (summary OR 1.64; 95% CI, 1.18-2.28) [42]. Nevertheless, the exact mechanism conferring the susceptibility to HT has not been elucidated yet and further studies are needed to determine which *CTLA-4* polymorphism is causative. 

Aside from being associated with HT, *CTLA-4* seems to be the major TAb susceptibility gene. A decade ago, a linkage of the CTLA-4 region to the presence of TAbs was demonstrated by a whole genome linkage analysis [46] and subsequently, *CTLA-4* was confirmed as a main locus for TAb status also in a larger data set [47, 48]. Evidence provided by our studies of Slovenian HT patients indicated a strong association between TAb production and the three *CTLA-4* SNPs, including -318C/T in the promoter, 49A/G and *CT60*. In patients carrying one of those three SNPs, evidently higher TPOAb concentrations were demonstrated in relation to the polymorphous *CTLA-4* gene, while the association with the TgAb concentrations was weaker [49, 50]. 

#### Protein Tyrosine Phosphatase Nonreceptor-Type 22 (PTPN22) Gene

*PTPN22* is the most recently identified immune-regulatory gene associated with the autoimmune thyroid disease, which is located on chromosome 1p13. PTPN22, which is predominantly expressed in lymphocytes, acts as a negative regulator of T-cell activation, much like CTLA-4. 1858C/T SNP of the *PTPN22* gene, resulting in arginine to tryptophan substitution at codon 620 (R620W), was demonstrated to be a risk factor for many autoimmune diseases. The mechanism is not clear since the disease predisposing T allele has been demonstrated to enable even more efficient inhibition of T-cell activation. Presumably, weaker T-cell signalling may lead to impaired thymic deletion of autoreactive T cells or an increased PTPN22 function may result in inhibition of regulatory T cells (Tregs), which protect against autoimmunity [51]. An early study in HT patients demonstrated a significant association with 1858C/T SNP (OR 1.77; 95% CI, 1.56-3.97) [52]. Afterwards, this observation was neither confirmed in German, Tunisian and Japanese populations [53-55] nor in Slovenian patients included in our study (unpublished data). In a small group of patients with both HT and autoimmune diabetes, T allele was determined in 50% compared with only 14% in healthy controls (OR 6.14; CI, 2.62-14.38) [56], however, in a yet another study estimating the same polymorphism this association was not confirmed [57]. Recently, 5 other *PTPN22* SNPs have been tested in Japanese patients, showing no relation with HT, but a novel protective haplotype containing those SNPs has been observed [58].

#### Thyroglobulin Gene

As discussed previously, Tg is an important thyroid specific antigen, also present in the circulation, which makes it an easy target of the autoimmune response. Gene for Tg is located on the chromosome 8q24 and linkage of this region with HT and autoimmune thyroid disease was first identified by a Japanese and an American whole genome studies [59, 60]. A subsequent fine mapping of this region exposed *Tg* gene as one of the major thyroid specific susceptibility genes, linked and associated with the autoimmune thyroid disease [61]. Later, different alleles of various microsatellite markers and different SNPs of *Tg* gene were related to HT, possibly affecting its expression, antigenicity, iodination, or binding to HLA. The association of *Tgms2* microsatellite marker in intron 27 with HT was confirmed in Japanese [62] as well as in Caucasian population [63]. Sequencing of human *Tg* revealed 14 SNPs among which four SNPs, including exon 10-12 SNP cluster and exon 33 SNP, were associated with HT [64]. However, this observation was neither confirmed in a larger data set of the United Kingdom Caucasian patients [65] nor in Chinese population, although in the later study one haplotype was significantly associated with HT and TgAb positivity [66]. 

#### Vitamin D Receptor Gene

Vitamin D, which acts *via *vitamin D receptor (VDR), possesses immunomodulatory properties and its deficiency has been implicated in the development of autoimmune diseases. Many immune cells express VDR, dendritic cells in particular, where VDR stimulation has been shown to enhance their tolerogenicity. Tolerogenic dendritic cells promote development of Tregs with suppressive activity and therefore peripheral tolerance [67]. *VDR* gene is located on the chromosome 12q12 and its polymorphisms have been related to different autoimmune disorders such as type I diabetes or Addison’s disease. A decade ago, the association between *VDR-FokI* SNP in exon 2 and HT has been identified [68] which was later confirmed in the observation of Taiwanese Chinese patients [69]. In the Croatian population *VDR* gene 3’ region polymorphisms were related to HT, possibly affecting VDR mRNA expression [70]. A significant relation has also been discovered between HT and both promoter and intron 6 gene polymorphisms of CYP27B1 hydroxylase, which is located on chromosome 12q13, catalysing the conversion of 25 hydroxyvitamin D_3_ to its active form [71]. 

#### Cytokine Genes and other Immune-Related Genes

Lately, several genes encoding different inflammatory cytokines have been studied in HT, some of them also influencing the severity of the disease. Interferon (IFN)-γ, produced by T-helper type 1 (Th1) cells, promotes cell-mediated cytotoxicity which underlies thyroid destruction in HT. T allele of the +874A/T *IFN-γ* SNP, causing the increased production of IFN-γ, was associated with severity of hypothyroidism in HT patients [72]. Higher frequency of severe hypothyroidism was also observed in patients carrying CC genotype of -590C/T *interleukin 4 (IL-4)* SNP, leading to a lower production of IL-4, one of the key Th2 cytokines which suppresses cell-mediated autoimmunity [73]. Gene polymorphism of transforming growth factor (TGF)-β, inhibitor of cytokine production, was also associated with HT. T allele of +369T/C SNP, leading to a lower secretion of TGF-β, was more frequent in severe hypothyroidism than in mild hypothyroidism [74]. Similarly, more severe form of HT was associated with -2383C/T SNP of gene for forkhead box P3 (FoxP3), an essential regulatory factor for the Tregs development [75]. Unlike the severity of hypothyroidism, the development of HT itself was associated with C allele of *tumor necrosis factor (TNF)-α* -1031T/C SNP. Namely, C-allele carriers present with higher concentration of TNF-α which acts as the stimulator of the IFN-γ production [76].

## THE ROLE OF FEMALE SEX AND REPRODUCTION

### Female Sex

As indicated by numerous epidemiological studies, females present with positive TAbs up to three times more often than males [7, 8, 11, 77-81]. The largest NHANES III study has shown that females were positive for TPOAbs and TgAbs in 17% and 15.2%, respectively, while males only in 8.7% and 7.6%, respectively [8]. According to the estimation provided by the study of Danish twins, the genetic contribution to TPOAb and TgAb susceptibility in females was 72% and 75%, respectively, while in males it was only 61% and 39%, respectively [23]. The possible explanation for high female predominance in thyroid autoimmunity might be associated with the X chromosome containing a number of sex and immune-related genes which are of key importance in the preservation of immune tolerance [82]. Increased immunoreactivity might therefore be related to genetic defects of the X chromosome, such as structural abnormalities or monosomy. Accordingly, a higher incidence of thyroid autoimmunity was reported in patients with a higher rate of X chromosome monosomy in peripheral white blood cells [83] or in patients with Turner’s syndrome [84]. Another potential mechanism of impaired immunotolerance in females is skewed X-chromosome inactivation (XCI) leading to the escape of X-linked self-antigens from presentation in thymus with subsequent loss of T-cell tolerance. Skewed XCI was associated with a higher risk of developing autoimmune thyroid diseases. Recently reported frequencies of skewed XCI in HT were 31%, 34.3%, 25.6% and 20%, respectively, which is significantly higher than in healthy controls, where the prevalences were only 8%, 8%, 8.6% and 11.2%, respectively [85-88]. Furthermore, a study of Danish twins demonstrated a significant association of skewed XCI with TPOAb serum concentrations in dizygotic but not in monozygotic twin pairs, indicating that shared genetic determinants of XCI pattern and TPOAb production are more likely than causal relationship [89].

### Pregnancy and Postpartum Period

The tolerance of the fetal semi-allograft during pregnancy is enabled by the state of immunosuppression which is a result of hormonal changes and trophoblast expression of key immunomodulatory molecules. The pivotal players in regulation of the immune response are Tregs, which rapidly increase during pregnancy. Consequently, both cell-mediated and humoral immune responses are attenuated with a shift towards humoral immune response, resulting in immune tolerance of the conceptus tissues and suppression of autoimmunity [90, 91]. Accordingly, the decrease of both TPOAb and TgAb concentrations during pregnancy has been reported, reaching the lowest values in the third trimester [91-94]. 

Postpartum rapid decrease of Tregs and re-establishment of the immune response to the pre-pregnancy state may lead to the occurrence or aggravation of the autoimmune thyroid disease [91]. The increase of TPOAb concentrations occurred as soon as 6 weeks after delivery [94], reaching the baseline level at approximately 12 weeks and the maximum level at about 20 weeks after delivery [92, 93]. In up to 50% of females with positive TPOAbs in the early pregnancy, thyroid autoimmunity in the postpartum period exacerbates in the form of postpartum thyroiditis. It may occur within the first year after delivery, usually clinically presented with transient thyrotoxicosis and/or transient hypothyroidism, while in about a third of females permanent hypothyroidism may even develop [95]. Interestingly, a significantly higher secretion of IFN-γ and IL-4 together with a lower median plasma cortisol concentration in 36^th^ week of gestation has been reported in females with postpartum thyroiditis than in euthyroid females, indicating that weaker immunosupression in the former group of females in late pregnancy could contribute to the postpartum thyroid dysfunction [96]. 

### Fetal Microchimerism 

The term fetal microchimerism is defined by the presence of fetal cells in maternal tissues which are transferred in the maternal circulation during pregnancy. Several years after the delivery, the chimeric male cells can be detected in the maternal peripheral blood [97, 98] as well as in maternal tissues, such as thyroid, lung, skin, or lymph nodes [99]. The fetal immune cells, settled in the maternal thyroid gland, may become activated in the postpartum period when the immunotolerance ceases, representing a possible trigger that may initiate or exaggerate the autoimmune thyroid disease. In HT, fetal microchimeric cells were detected in thyroid in 28% to 83% [100-103] which means that their occurrence is significantly higher than in the absence of autoimmune thyroid disease. Furthermore, a recent study of twins supported the putative role of microchimerism in triggering thyroid autoimmunity, showing a significantly higher prevalence of TAbs in opposite sex twins compared to monozygotic twins [104]. Additionally, euthyroid females having been pregnant presented significantly more often with positive TPOAb compared to females with no history of being pregnant [105]. However, the relation between parity and autoimmune thyroid disease was not confirmed by large population-based studies, advocating against the essential contribution of fetal microchimerism to the pathogenesis of autoimmune thyroid disease [106-109].

## ENVIRONMENTAL TRIGGERS

### Iodine Intake

Excessive iodine intake is well-established environmental factor for triggering thyroid autoimmunity. Several large population-based studies demonstrated higher prevalence of TAbs in the areas with higher iodine supply since the estimated prevalence was approximately 13% in iodine deficiency [7], 18% in circumstances of sufficient iodine intake [8] and about 25% in areas with excessive iodine intake [11]. Moreover, up to four-fold increase in prevalence of TAbs was demonstrated after the exposure to higher iodine intake due to the improvement of iodine prophylaxis in previously iodine deficient areas [110-112]. According to the intervention study, deliberate exposure to 500 μg of iodine provoked thyroid autoimmunity in 20% of previously healthy individuals [113]. Valuable evidence was also provided by using experimental animal models of autoimmune thyroiditis, where the prevalence and severity of thyroid autoimmunity significantly increased when the dietary iodine was added [114]. 

Several putative mechanisms by which iodine may promote thyroid autoimmunity have been proposed. Firstly, iodine exposure leads to higher iodination of Tg and thus increases its immunogenicity by creating novel iodine-containing epitopes or exposing cryptic epitopes. This may facilitate presentation by APC and enhance the binding affinity of the T-cell receptor which may lead to specific T-cell activation [114]. Secondly, iodine exposure has been shown to increase the level of reactive oxygen species in the thyrocyte which is generated during TPO oxidation of excessive amounts of iodine. They enhance the expression of the intracellular adhesion molecule-1 (ICAM-1) on the thyroid follicular cells which could attract the immunocompetent cells into the thyroid gland [115]. Thirdly, iodine toxicity to thyrocytes has been reported, since highly reactive oxygen species may bind to membrane lipids and proteins, causing thyrocyte damage and release of autoantigens [112]. Fourthly, iodine excess has been shown to promote follicular cell apoptosis by inducing an abnormal expression of tumor necrosis factor-related apoptosis-inducing ligand (TRAIL) and its death receptor (DR)-5 in thyroid [116]. Fifthly, *in vitro* evidence also suggests an enhancing influence of iodine on the cells of the immune system, including augmented maturation of dendritic cells, increased number of T cells and stimulated B-cell immunoglobulin production [112]. 

### Drugs

Furthermore, certain drugs were reported to trigger or exacerbate thyroid autoimmunity in susceptible individuals. Interferon α (IFN-α) is extensively used to treat chronic hepatitis and is frequently associated with thyroid autoimmunity since TAbs were observed in up to 40% and clinical disease in 5-10% of patients treated with IFN-α. Presumably, IFN-α has both thyroid toxic effects with consequent autoantigen presentation and immune effects, such as switching to Th1 immune response, suppression of Treg function, activation of immune cells, stimulation of cytokine release and expression of MHC class I on thyroid cells [117]. Similarly, IL-2 treatment, used for melanoma and renal carcinoma, seems to act *via *immune and toxic mechanisms, leading to both TAb positivity and hypothyroidism [118]. In patients with known autoimmune thyroid disease lithium may increase the risk of hypothyroidism. According to some studies, treatment with lithium has also been shown to increase TAb titres and the prevalence of thyroid autoimmunity, although this observation has not yet been confirmed by other reports [118, 119]. Among putative mechanisms direct toxicity of lithium on thyroid or toxicity of increased intrathyroidal iodine resulting from lithium treatment were discussed [118]. Similarly, amiodarone alone as well as its high iodine content may act cytotoxically which may lead to thyroid autoantigen presentation and provoke thyroid autoimmunity [120]. 

### Infections

Not only the IFN-α treatment but also hepatitis C infection itself has been reportedly associated with thyroid autoimmunity and hypothyroidism. Among possible mechanisms, the molecular mimicry between viral and self-antigens has been suggested, whereas the release of pro-inflammatory mediators caused by viral infection may lead to activation of autoreactive T-cells [117]. Besides, in HT several other putative triggering viruses have been implicated such as parvovirus, rubella, herpes simplex virus, Epstein Barr virus, and human T-lymphotropic virus type 1 [121]. A recent study of sera in pregnant women has also indicated an association between a prior infection with Toxoplasma gondii and an increase of TPOAbs [122]. Nevertheless, the evidences are scarce and further studies are required in order to confirm the role of infections as causative agents. 

### Chemicals

The exposure to environmental toxicants such as polyaromatic hydrocarbons or polyhalogenated biphenyls, both commonly used in a variety of industrial applications, has been shown to provoke thyroid autoimmunity not only in experimental animals but also in humans [115]. Recently, a significantly higher prevalence of HT and TAb (9.3% and 17.6%, respectively) has been demonstrated in residents living in the area of petrochemical complex of Sao Paolo compared to the control area (3.9% and 10.3%, respectively) [123]. In Slovakia, the exposure to polychlorinated biphenyls was associated with TAb and hypothyroidism [124]. Although there is strong evidence attesting the contribution of chemicals to thyroid autoimmunity, the exact mechanisms of their action are yet to be established.

## PROTECTION AGAINST THYROID AUTOIMMUNITY

Two mechanisms enable the maintenance of self-tolerance. The central tolerance refers to thymic deletion of autoreactive T cells during fetal life. Those cells that escape central tolerance are prevented from triggering autoimmunity by mechanisms of peripheral tolerance where the pivotal role is played by Tregs [91, 125]. They have a suppressive effect on the effector T cells, APCs and B cells, therefore maintaining the immunological unresponsiveness to self-antigens and suppressing the excessive immune response [126]. They may directly suppress target cells or act through secreted suppressor cytokines. 

Tregs derive from thymus as a subpopulation of T cells or from naive T cells in the periphery and express CD25 (α chain of the IL-2 receptor) and FoxP3. Therefore, the critical role in the immune system is played by CD4^+^CD25^+^Foxp3^+^ Tregs [125]. Cells with the highest CD25 expression (CD4+CD25high) are responsible for regulatory suppressive effects [127]. The role of Tregs in self-tolerance was extensively studied in animal models, especially on murine experimental autoimmune thyroiditis, where the protection against autoimmunity was mediated by thymically-derived CD4^+^CD25^+^Foxp3^+^ Tregs [128]. In humans, CD25 expression was higher in patients with HT than in healthy subjects [129]. When patients with autoimmune thyroid disease were studied, the proportion of Tregs was lower intrathyroidally than in peripheral blood [130]. When compared to controls, a higher proportion of special Tregs with high levels of FoxP3 (termed CD4+CD25highHLA-DR+ cells) in HT patients was found by three-color flow cytometry [131]. This indicates a compensatory expansion of Tregs subpopulation in order to diminish the immune response [132]. 

The expression of Foxp3 and generation of Treg cells are both induced by TGF-β produced in Tregs, fibroblasts, macrophages, endothelial cells in inflammatory thyroid tissue as well as in thyrocytes [133, 134]. TGF-β is a key regulator of immune tolerance which stimulates suppressive Tregs and inhibits T cell differentiation [134]. Accordingly, in patients with HT, serum levels of TGF-β were lower than in controls which did not change after the treatment with levothyroxine. Therefore, levels of TGF-β seem to be associated with the HT and not thyroid dysfunction [135]. Suppressive effect of TGF-β was established in the development of experimental autoimmune thyroiditis [136]. At the later stage of the thyroiditis TGF-β may trigger the development of fibrosis [137]. 

## DEVELOPMENT OF THYROID AUTOIMMUNITY

For the initiation of thyroid autoimmunity the proper interaction of thyroid cells, APCs, and T cells is necessary. The result is a secretion of different cytokines leading to Th1 response which is characteristic of HT. Cytokines have an important role in autoimmune thyroid disease and are secreted by the immune cells, thyroid follicular cells, and inflammatory cells [138]. In spite of the extensive researches in last decades, the exact mechanisms of initiation and progression of thyroid autoimmunity have not been completely clarified yet. 

T lymphocyte proliferates and secretes cytokines after being stimulated by two signals. First signal is the activation of T-cell receptor which is induced by binding of antigen peptide presented on HLA molecule on the surface of APC. Second costimulatory signal is ligation of CD28 on the surface of T cell to B7 ligands (B7.1 and B7.2) expressed on APC, including dendritic cells, activated macrophages, and activated B cells. As explained previously, activation of T cell induces the expression of CTLA-4 which suppresses further T cell activation by binding and blocking of B7 ligands. In patients with HT, but not in GD or goitrous patients, thyroid follicular cells were positive for B7.1 [139]. Thyroid follicular cells may also express MHC class II molecules induced by IFN-γ which is secreted by intrathyroidal lymphocytes [140]. Colocalization of MHC class II and B7.1 antigens on the same thyroid follicular cell indicates that in the pathogenesis of HT follicular cells may act also as APCs and may be capable of inducing or maintaining the autoimmune process. Besides, the interaction between thyroid follicular cells and lymphocytes seems to be crucial for the progression of thyroid autoimmunity [140]. 

**Fig. (1):**
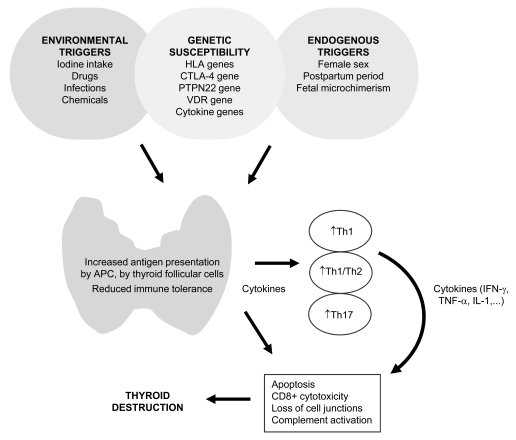
Mechanisms of thyroid autoimmunity in HT. In individuals with certain genetic background, several endogenous and environmental factors may trigger thyroid autoimmunity, causing increased antigen presentation in the thyroid and consequently leading to reduced immune tolerance. As a result, different cytokines are produced by immune and thyroid cells leading to predominantly Th1 response with increased Th1/Th2 ratio. Lately, also Th17 effector T cells have been implicated in thyroid autoimmunity. Increased production of cytokines, such as interferon γ (IFN-γ), tumor necrosis factor α (TNF-α), and interleukin 1 (IL-1), may lead to apoptotic processes which combined with CD8+ mediated cytotoxicity, impairment of cell junctions, and complement activation slowly induce thyroid destruction.

Autoimmune response is predominantly determined by the antigen, nature of APC, and several genetic, endogenous, and environmental triggers [140]. In HT, where possible triggers were previously discussed, autoimmune response is mediated predominantly by Th1-type cytokines such as TNF-α, IFN-γ and IL-2 which all participate in the pathogenesis in a harmful way (Fig. **1**) [141]. According to cytokine mRNA profiles, both Th1 and Th2 response were supposed to be involved in the pathogenesis of HT with deviation toward Th1 pattern [141]. When exposed to a thyroid self-antigen such as human Tg, peripheral blood mononuclear cells from patients with HT produced more TNF-α, IL-2, IL-10, IFN-γ, and less IL-5 than controls. Since higher production was observed with autologous sera than with pooled normal sera, the importance of serum elements, such as the complement and serum anti-Tg activity, was proposed. Authors postulated that these two serum elements accelerate Th1/Th2 cell cytokine reaction by increasing the uptake of autoantigens by APCs [142]. Interestingly, peripheral Th1/Th2 ratio seems to also be related to the severity of HT since patients with more severe hypothyroidism presented with a higher ratio compared with those with mild hypothyroidism [143]. Furthermore, the correlation between the TPOAb levels and the production of TNF-α and IFN-γ has been demonstrated [144] and recently confirmed by a study indicating that TPOAbs seem to promote TPO-elicited cytokine production, including IFN-γ, TNF-α, and IL-6 [145]. 

In the past few years, a new subset of Th cells, designated Th17, has been studied. Th17 cells have also been proved highly proinflammatory and may lead to autoimmunity in animal models and potentially also in human autoimmune diseases [134]. A putative importance of Th17 in the pathogenesis of HT has been indicated by a recent study showing increased levels of Th17 lymphocytes and Th17 cytokines compared with healthy controls [146]. Similarly, when estimating the expression in peripheral blood mononuclear cells, Th17 cells rather than Th1 cells predominated in HT patients [147].

## THYROID DESTRUCTION

Thyroid destruction in HT is mostly a consequence of the apoptotic processes combined with CD8+ cell mediated cytotoxicity, changes in cell junctions, and complement activation. 

Apoptosis is characterized by cytoskeletal disruption, cell shrinkage, chromatin condensation, nuclear fragmentation, membrane blebbing, and DNA fragmentation [148]. In general, apoptosis enables adult healthy tissue homeostasis, while excessive cell death causes a loss of active tissue and an impaired function [148]. Two major apoptotic signal pathways have been studied more thoroughly [149]. The first pathway consists of Fas receptor (Apo-1 or CD95), a type I transmembrane protein and a member of TNF receptor superfamily, and Fas ligand, a type II transmembrane protein which binds to Fas receptor and activates apoptotic process [150]. The second pathway includes TRAIL (Apo2L), a member of TNF receptor family which activates apoptosis by binding to its receptors TRAIL-R1 (DR4) and TRAIL-R2 (DR5) [151, 152]. Both pathways lead to the activation of proteolytic enzymes caspases which destroy thyroid cells. In normal circumstances, the Fas pathway is inhibited, whereas in inflammation, it is activated by cytokines [153]. *In vitro *studies have shown that TSH inhibited Fas-mediated apoptosis, while the absence of TSH induced apoptosis [154]. A combination of IFN-γ with TNF-α or IL-1β stimulated the Fas-activated apoptosis in cultured thyroid follicular cells [155]. TRAIL receptors are also expressed in thyroid cells by cytokines [156]. TRAIL could mediate the programmed cell death under appropriate conditions [157]. Thyroid follicular cells were able to express TRAIL in the presence of IFN-γ, TNF-α, and IL-1β *in vitro*. Thus, inflammatory cytokines such as IFN-γ, TNF-α, and IL-1 can influence immune-mediated apoptosis [156]. Additional inductor of programmed cell death is TGF-β [158]. Its release from thyroid cells is stimulated by the epidermal growth factor (EGF) [159]. In porcine thyroid follicles both TGF-β and EGF induced apoptosis, the latter probably also by its influence on TGF-β expression [160]. Therefore, the mechanism of apoptosis in HT seems to be induced by cytokines secreted from local lymphocytes [161]. 

In HT, the main apoptotic mechanism represents Fas-mediated apoptosis together with downregulation of Bcl-2 which is an inhibitor of apoptosis highly expressed in normal thyrocytes [162-165]. Bcl-2 immunostaining was high in healthy thyroid follicles and in follicles away from lymphocytic infiltrates in HT, but weak in thyroid follicles surrounded by lymphocytic infiltrates in thyroid glands with HT [163]. Immunohistochemically, patients with HT had a lower percentage of Bcl-2 stainings and a higher percentage of caspase-3 reactions than control subjects [166]. In thyroid glands with HT, a strong staining for Fas and Fas ligand and a high percentage of apoptosis was observed in thyroid follicles adjacent to lymphocytes when compared with healthy thyroid tissue where a moderate, minimal, or no Fas ligand and almost no apoptosis were found. In HT, there was also a higher number of CD8+ cells expressing Fas found than in healthy subjects [164]. 

Beside apoptosis, CD8+ cell mediated cytotoxicity contributes to thyroid destruction mediated by ICAM-1 which is expressed by thyroid follicular cells and increases CD8+ cell binding [140]. Levels of ICAM-1, vascular cell adhesion molecule-1 (VCAM-1), and tissue inhibitor of metalloproteinases 1 (TIMP-1) were significantly higher in patients with functional abnormality due to the autoimmune thyroid disease than in euthyroid subjects. Authors postulated that autoimmune process itself may have an impact on vascular inflammation, endothelial dysfunction, and tissue remodelling [167]. 

Finally, changes in gap junctions consequently leading to cell-to-cell communication changes may contribute to the thyroid destruction. In HT, decreased levels of connexin 43 (Cx43) were observed immunohistochemically when compared with the normal thyroid tissue [168]. Cx43 is a part of connexons which constitute a gap junction channel in organized tissues as well as in thyroid tissues. Similarly, reduced proteins Cx43, Cx32, and Cx26 were established in animal models of autoimmune thyroid disease [169]. Studies also showed that T_3_ increased the level of Cx43 [170]. Therefore, the number of gap junctions may be increased in GD and decreased in HT [168]. Disruption of thyroid integrity may allow TPO antibodies to bind to TPO and activate complement [140]. 

## CONCLUSION

HT is one of the most prevalent autoimmune diseases provoked in genetically susceptible individuals by several triggers, including female sex, immune changes after delivery, fetal microchimerism, iodine intake, and other environmental factors. Multiple susceptibility genes may be involved in the disease development, some of which are also common for other autoimmune diseases, while others are specific for thyroid autoimmunity. It is now clear that immune-regulatory genes such as *HLA*, *CTLA-4*, and *PTPN22* play a major role in the aetiology of HT, GD, and several other autoimmune diseases. The only thyroid specific gene currently showing the association with HT is gene for Tg which is also GD susceptibility gene. *VDR* gene is another HT predisposing gene, common for other organ-specific autoimmune diseases such as type I diabetes or Addison’s disease. Furthermore, recent studies of cytokine genes such as *IFN-γ*, *IL-4*, or *TGF-β* indicate the association with the development and severity of HT, presumably influencing the balance between Th1 and Th2 mechanisms. 

According to the current knowledge, a complex interaction between genetic and non-genetic factors presumably results in enhanced thyroid antigen presentation and reduced immune tolerance leading to predominantly Th1-type autoimmunity, thyroid destruction, and clinical disease. Despite tremendous progress made in the understanding of HT during the past decade, the exact mechanisms of its progression are yet to be clarified. Hopefully, in the near future, new evidence will enable a better insight into the disease pathogenesis which may help us identify subjects at risk and may be even enable us to prevent the development of clinical disease. 

## CONFLICTS OF INTEREST

The authors have no conflicts of interest to declare.

## ABBREVIATIONS

APC: = Antigen presenting cell
CTLA-4: = Cytotoxic T lymphocyte antigen-4
DR: = Death receptor
FoxP3: = Forkhead box P3
GD: = Graves’ disease
HLA: = Human leukocyte antigen
HT: = Hashimoto’s thyroiditis
ICAM-1: = Intracellular adhesion molecule-1
IFN: = Interferon
IL: = Interleukin
MHC: = Major histocompatibility complex
NHANES: = National Health and Nutrition Examination Survey
PTPN22: = Protein tyrosine phosphatase nonreceptortype 22
SNP: = Single nucleotide polymorphism
TAbs: = Thyroid autoantibodies
T_4_: = Thyroxine
Tg: = Thyroglobulin
TgAbs: = Antibodies against Tg
TGF: = Transforming growth factor
TIMP-1: = Tissue inhibitor of metalloproteinases 1
Th: = T-helper
TNF: = Tumor necrosis factor
TPO: = Thyroid peroxidase
TPOAbs: = Antibodies against TPO
TRAIL: = Tumor necrosis factor-related apoptosis-inducing ligand
Tregs: = Regulatory T cells
T_3_: = Triiodothyronine
UTR: = Untranslated region
VCAM-1: = Vascular cell adhesion molecule-1
VDR: = Vitamin D receptor
XCI: = X-chromosome inactivation
